# Sex and Stress Steroid Crosstalk Reviewed: Give Us More

**DOI:** 10.1210/jendso/bvaa113

**Published:** 2020-09-07

**Authors:** Jan Kroon, Onno C Meijer

**Affiliations:** 1 Department of Medicine, Division of Endocrinology, Leiden University Medical Center, ZA, Leiden, the Netherlands; 2 Einthoven Laboratory for Experimental Vascular Medicine, Leiden University Medical Center, ZA, Leiden, the Netherlands

**Keywords:** glucocorticoids, nuclear receptor, sexual dimorphism, sex hormones

For many diseases, the incidence, severity, and/or symptoms differ between males and females. This holds true, for example, for autoimmune diseases (more prevalent in females) and cardiometabolic diseases (typically more common in males). In addition to the obvious chromosomal differences, sex hormones, such as the predominantly male testosterone and the predominantly female estradiol, contribute to sex-dependent disease development. However, the paradigm of “male and female” hormones is too simplistic. Intracellular testosterone-to-estradiol conversion (by the enzyme aromatase) plays a key role in male physiology [1], while androgen signaling is prominent in many tissues in females [2]. To add to this complexity, evidence is accumulating that *crosstalk* between sex steroid hormones and glucocorticoid stress hormones substantially contributes to sex-dependent physiology and disease [3].

The importance of this steroid hormone crosstalk was comprehensively described in a recent publication by Daniel Ruiz, Vasantha Padmanabhan, and Robert Sargis [4]. In their review, the authors focus on metabolic (mis)programming during fetal development, and discuss that early overexposure to glucocorticoids (either exogenous, or as a consequence of stress) can disrupt sex steroid action later in life. Also in adult tissues, there is clear evidence for extensive crosstalk at a molecular (e.g., interactions between steroid receptors), enzymatic (e.g., synthesis and breakdown of hormones) and functional level.

Glucocorticoid-sex steroid crosstalk can be—and often is—bidirectional [5]. This is intrinsic to direct receptor interactions, but also may involve hormone metabolism, mutual effects on the brain-pituitary axes that drive steroid secretion, and yet more indirect mechanisms. The sex-dependency in glucocorticoid outcome can be quantitative (a glucocorticoid-induced effect is present in both sexes but is stronger in males or females) or qualitative (“all or nothing,” e.g., a robust upregulation of particular glucocorticoid-responsive genes, specifically in males or females, but not in the other sex) [6].

While much research focuses on androgens and estrogens, progesterone receptor signaling is often ignored but may in fact contribute to sex-differences in metabolism as well, as covered by Ruiz et al [4]. In fact, steroid hormone receptors—glucocorticoid, androgen, progesterone, and the mineralocorticoid receptors; and to a lesser extent the estrogen receptor-α and -β—are very similar in molecular structure. While small differences between the steroid hormones and between their receptors lead to remarkable specificity, it should come as no surprise that their transcriptional targets are often not specific for one nuclear receptor and that ligands/receptors can be promiscuous. Indeed, there is good evidence for heterodimerization between nuclear receptors, but the quaternary structure of nuclear receptor complexes may in fact involve tetramers or even more complicated transcriptional complexes. As an example of a more indirect manner of crosstalk, transcriptional activity of the glucocorticoid receptor has been shown to be modulated by follicle stimulating hormone [7]. Functionally, crosstalk is thus not necessarily limited to the steroidal hormones.

In the current research landscape, steroid hormone crosstalk is often overlooked despite the fact that many (metabolic) processes are sex-dependent. For example, in the field of endocrinology, as little as 10% of animal studies include both males and females, although fortunately this percentage is much higher in studies with human subjects (>50%) [8]. An argument that is often used in animal research is that the rodent 3- to 5-day estrous cycle creates more variation in experimental outcomes in females, and that much higher numbers of female animals are therefore required to generate statistically robust data. While this may be true for short-term studies, many longer experiments cover multiple full cycles making it unlikely to substantially influence long-term (longitudinal) outcomes, and it is possible to control for the estrous cycle phase for short-term studies. It should therefore be relatively easy to bypass the constraint that the estrous cycle evokes too much variation in many experimental settings.

It makes perfect sense that scientific societies and funding bodies advocate the use of both males and females in biomedical research—which is nowadays endorsed by many (e.g., Dutch Heart Foundation, NWO-Dutch Science Agenda, National Institutes of Health). We are pleased that the current review by Ruiz and colleagues [4] puts additional focus on the importance of sex-dependency and steroid hormone crosstalk, and points out the many underresearched aspects of sex differences in general, and steroid crosstalk in particular.

## Additional Information

***Disclosure Summary:*** The authors have nothing to disclose.
